# Introgression and Potential Outbreeding Depression Following Anthropogenically‐Induced Secondary Contact Between Anadromous and Resident Atlantic Salmon ( *Salmo salar* )

**DOI:** 10.1111/eva.70318

**Published:** 2026-09-25

**Authors:** C. S. T. MacPhail, T. Kess, I. A. Fleming, N. Smith, I. R. Bradbury

**Affiliations:** ^1^ Ocean Sciences Centre, Memorial University of Newfoundland St. John's Newfoundland and Labrador Canada; ^2^ Northwest Atlantic Fisheries Centre, Fisheries and Oceans Canada St. John's Newfoundland and Labrador Canada

**Keywords:** ecotypes, intraspecific diversity, partial migration, population genomics, salmon enhancement, stocking

## Abstract

The evolution of alternative migratory strategies can lead to the establishment of ecologically and genetically distinct morphs; however, the forces that maintain these differences are often unclear. Here we assessed how migratory strategy may affect gene flow and adaptation in Atlantic Salmon ( *Salmo salar* ) in a setting where a migratory barrier was removed, and migratory and non‐migratory salmon have been brought into contact through a program to initiate a migratory population. We used whole genome sequencing to characterize genetic variation in resident and anadromous salmon in this experimental system, as well as in the original source of the migratory individuals and a proximate source of strays into the system. Our results suggest that resident and anadromous salmon populations are highly divergent genetically, but that introgression from the resident population into the introduced migratory morph is widespread. However, despite a general trend of introgression into the migratory form, localized genomic regions appear resistant to gene flow, potentially highlighting adaptive divergence between resident and anadromous salmon. Additionally, we compared how the proportion of ancestry associated with migratory type changed between out migrating smolts and returning adults across two cohorts. Our results show a significant reduction in resident ancestry consistent with outbreeding depression during the marine migratory phase. Genomic comparisons between smolts and adults also identify several localized regions potentially under selection in the marine environment, possibly related to immune function. Together, these results suggest that introgression between divergent migratory forms in this system is unidirectional and is resulting in outbreeding depression and reduced migratory success. In the context of supplementation, our results suggest that enhancement activities aimed at boosting anadromous Atlantic Salmon populations could be impacted by introgression with resident populations. Additionally, interbreeding may present a demographic threat to resident populations, as anadromous‐resident hybrids appear to follow a migratory life history.

## 1 Introduction

Intraspecific variation is directly related to a species' ability to adapt and persist in the face of ecological change (Des Roches et al. 2018, 2021). Alternative migratory strategies may play an important role in generating intraspecific diversity, but this role is currently poorly understood. Many migratory species vary in when, where, and if they migrate among populations and even individuals (Chapman et al. 2011; Dodson et al. 2013). As a result, individuals of the same species with differing migratory strategies may be exposed to vastly different environments, physiological demands and biotic interactions, which apply distinct selective pressures (Davis et al. 2017). In this way, alternative migratory strategies could result in divergent plastic and genetic adaptations (Rolshausen et al. 2009, Ruegg et al. 2014, Lundberg et al. 2017, Ferguson et al. 2019, Tigano and Russello 2022), increasing intraspecific variation. However, the extent to which this process can cause divergent adaptation, and consequently affect gene flow and reproductive isolation, remains unclear in most instances.

Atlantic Salmon ( *Salmo salar* ) (Linnaeus, 1758) are a culturally and economically important salmonid fish (Myrvold et al. 2019) native to the North Atlantic that display a myriad of migratory phenotypes (Hutchings et al. 2019). Perhaps the most divergent of these migratory phenotypes are those that migrate from their natal streams to the ocean (anadromous) and those that complete their entire life cycle in freshwater (resident). While resident Atlantic salmon are uncommon in the species' European range, they are widely distributed across its North American range and are especially common on the island of Newfoundland (Hutchings et al. 2019). Studies comparing genetic divergence between proximate populations of anadromous and resident Atlantic salmon have found variable results, ranging from little to substantial divergence depending on the location (Perrier et al. 2013; Zueva et al. 2014; Adams et al. 2016; Kjærner‐Semb et al. 2021; Harder and Christie 2022; Hindar et al. 2025). Adding to the confusion, much of this divergence is likely to be caused by genetic drift, which can be comparatively intense in resident populations as they tend to be smaller and isolated, making it hard to detect signatures of natural selection (Perrier et al. 2013; Zueva et al. 2014). More recent studies using genomic methods have identified evidence of parallel divergent selection between many populations of resident and anadromous Atlantic salmon, suggesting that migratory strategy does play a role in generating adaptive genetic diversity in this species (Zueva et al. 2014; Kjærner‐Semb et al. 2021; Harder and Christie 2022). Genomic tools may be particularly useful in clarifying the level and nature of adaptive divergence driven by alternative migratory strategies in Atlantic salmon, and the potential ecological and demographic consequences of changes to this diversity across ecological contexts.

Atlantic salmon populations have declined and, in some cases, disappeared across the species' range in recent years (Lehnert et al. 2019). This is increasingly prompting consideration of enhancement and supplementation action, such as stocking (Lennox et al. 2021). Interactions among migratory morphs brought together by enhancement and supplementation activities could both illuminate the underpinning evolutionary forces and genomic architecture, as well as hamper the effectiveness of these conservation measures and/or threaten the unique intraspecific diversity contained in resident populations. For example, recent work suggests that stocking of anadromous salmon into areas with resident forms can result in introgression and threaten the persistence of the resident form, as hybrids seem to display a preference to migrate (Hindar et al. 2025). What remains unclear is the degree to which selection is shaping the genomic variation present during introgression; if so, gene flow between adaptively differentiated anadromous and resident populations could be maladaptive. Assessing the degree of adaptive differentiation between resident and anadromous morphs and whether maladaptive introgression results where they are brought into contact will inform future salmon enhancement measures and clarify the evolutionary novelty of resident populations.

**FIGURE 1:**
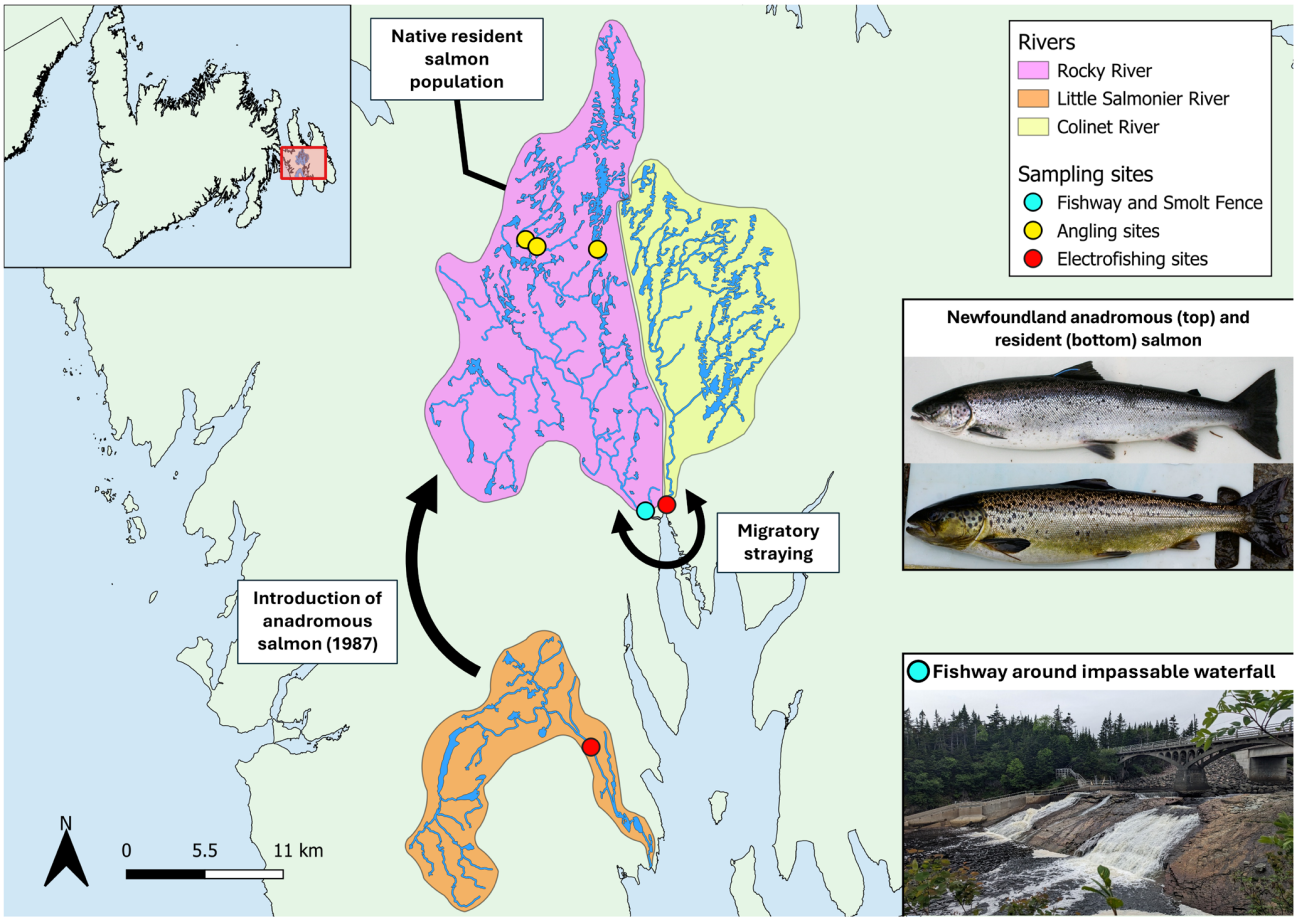
Map highlighting the study area, focal rivers, sampling sites and demographic relationships between the studied populations of Atlantic salmon ( *Salmo salar* ). Arrows and labels describe the history of population interactions. The impassable waterfall that historically prevented salmon migrations in the Rocky River and the fishway that now circumvents it are pictured on the bottom right. A comparison of adult Newfoundland anadromous and resident salmon is pictured on the centre right.

Here, our aim was to understand the genomic and fitness consequences of introgression between divergent migratory forms in a wild population. To this end, we take advantage of a natural experiment which put resident and migratory salmon into contact following an intensive salmon enhancement program at the Rocky River, Newfoundland, Canada. The program involved the construction of a fishway around an impassable waterfall and the stocking of millions of anadromous salmon fry from the Little Salmonier River beginning in 1984 (Bourgeois 1998) into a watershed previously solely occupied by the resident form (Figure 1). Specifically, our objectives were to use whole‐genome sequencing to (1) compare genetic variation in sympatric resident and anadromous Atlantic salmon from the Rocky River, Newfoundland, Canada, as well as anadromous salmon from two other nearby rivers including the source of the introduction; (2) explore the degree of introgression which has occurred in the three decades following the introduction; and (3) compare out‐migrating juvenile salmon (smolts) to returning migratory adults to explore genomic differences and the potential fitness consequences through outbreeding depression associated with the marine phase. This study directly builds on previous work documenting hybridization among migratory forms in this system (Mason 2018), comparing the genomics of migratory forms across the range (Zueva et al. 2014; Kjærner‐Semb et al. 2021; Harder and Christie 2022), and exploring the consequences of genetic introgression between migratory forms following supplementation (Hindar et al. 2025).

## 2 Methods

### 2.1 Study System

The Rocky River is a 296 km^2^ watershed located on the Avalon Peninsula in Southeast Newfoundland, Canada. The watershed historically contained only resident Atlantic Salmon as an impassable waterfall at the river mouth prevented anadromous behaviour (Bourgeois 1998). Anadromous salmon were introduced as part of a salmon enhancement project beginning in 1984; a functional fishway was constructed around the impassable barrier and broodstock derived from the Little Salmonier River population was stocked into the watershed. Across four successive years, an average of 260 Little Salmonier adult salmon (range 202–365; total 1040) were used to generate an average of 334,250 fry stocked per year (range 194,000–435,000; total stocking of 1,337,000 fry) (Bourgeois 1998; Hustins 2004). The Little Salmonier River, Rocky River resident, and the nearby Colinet River populations likely all derive from the same post‐glacial colonizers (Nugent et al. 2024), with the Rocky River residents having been isolated for likely several thousand years following the emergence of the impassable waterfall.

### 2.2 Sampling, DNA Extraction, and Sequencing

Genetic tissue samples were collected using several approaches from both the Rocky River and adjacent watersheds. We sampled 44 resident adult salmon from three lakes on the Rocky River watershed using angling gear in 2024 and obtained 64 smolt and 126 anadromous adult samples collected from the Fisheries and Oceans Canada (DFO) counting facilities at the mouth of the Rocky River in 2022–2024. Smolt and anadromous adults were sampled as part of DFO sampling programs from a smolt fence and fishway at regular intervals across the breadth of their respective migratory seasons. We also sampled 61 parr and fry from a site on the Colinet River and 48 parr and fry from a site on the Little Salmonier River in 2024 by electrofishing.

Captured individuals large enough to non‐lethally sample were anaesthetized using either clove oil (Rocky River adult resident, all Colinet and Little Salmonier individuals) or MS‐222 (Rocky River smolts and adults) before measuring, weighing, and taking a fin clip from the individual. Fin clips were immediately placed in Eppendorf tubes containing 95% ethanol. Sampled individuals were placed in a recovery bucket with an airstone until they regained equilibrium and then released shortly thereafter. Individuals that were too small to sample non‐lethally (primarily young‐of‐the‐year) were euthanized with a higher dose of clove oil before sampling. Carcasses of these individuals were then frozen for future analyses.

We conducted low‐coverage (~4× depth) whole genome sequencing of our samples. DNA was extracted using the DNeasy 96 Blood and Tissue kit (Qiagen) following the manufacturer's protocol. DNA quality was assessed on a 1% agarose gel and concentration was measured using Qubit HS dsDNA quantification kit (ThermoFisher). Samples were normalized to equimolar concentrations and then prepared using DNA Prep kit (Illumina). Individual libraries were pooled and had their size quantified on a 2100 Bioanalyzer (Agilent Technologies). Libraries were then sequenced on an Illumina NovaSeq6000 S4 at the Genome Quebec Centre d'Expertise et de Services.

### 2.3 Bioinformatic Processing

Raw sequence reads were genotyped using a modification of the pipeline used in Kess et al. (2024), briefly described here. We checked the quality of sequence reads using FastQC (Andrews 2010) and then used *fastp* 0.23.2 (Chen et al. 2018) with the “‐cut_right” modification with a window step of 4 and a quality score cutoff of 20 to trim the sequences. This step removes low quality reads and polyG adapter sequences. We then aligned the remaining, trimmed reads to the Ssal_v3.1 *Salmo salar* reference assembly (GCF_905237065.1) using *bwa mem* 2 (Vasimuddin et al. 2019). We then used the Genome Analysis Toolkit (GATK 4.4.0, McKenna et al. 2010) to remove duplicated reads using the MarkDuplicates function and then sorted and used GATK 3.8 to realign the remaining reads around potential insertions and deletions using the RealignerTargetCreator and IndelRealigner functions. We then simultaneously genotyped samples and exported genotype likelihoods using ANGSD v 0.940 with parameters described in Kess et al. (2024). We used high per base and alignment thresholds to remove low quality alignments and genotype calls (Q > 30) and set a minimum of 2× read depth and 80% genotyped threshold. Following genotyping, we removed individuals and loci that lacked more than 25% of the genotype information. We also conducted a preliminary principal components analysis (PCA) in PLINK (version 1.9, Chang et al. 2015) to identify individuals that were Brown Trout ( *Salmo trutta* ) (Linnaeus, 1758) misidentified as Atlantic salmon parr (*n* = 2). We then imputed the data using Beagle 4.0 (Browning and Browning 2007) under default parameters. We compared allele frequencies, PC1, and PC2 scores between the imputed data and the likelihood data from ANGSD for Chromosome 28 from all the samples in our dataset. These metrics were > 99% correlated (*p* = −2.2e16) between the two datasets (Figure S4), suggesting that both datasets are capturing the same genomic patterns as expected with > 4× read depth in a system with high linkage disequilibrium like Atlantic Salmon (Lou et al. 2021). We then filtered the dataset in PLINK to remove alleles with a frequency of less than 0.01. We also created a linkage disequilibrium (LD) pruned dataset by using the ‐‐indep‐pairwise command in PLINK to identify SNPs in linkage disequilibrium and then remove them, using 50 SNP windows with a sliding window of 5 and *r* ^2^ greater than 0.5. Lastly, we used KING 2.0 (Gao and Chen 2017) to evaluate relatedness and identify close kin in our samples that might skew population structure analyses. No first‐degree relatives were detected.

### 2.4 Characterizing Genetic Variation Among Populations

To understand the broad‐scale genomic relationships among our studied populations, we first ran a PCA of the imputed whole‐genome data across all populations in PLINK (version 1.9, Chang et al. 2015). We also ran the ADMIXTURE program (v1.3, Alexander et al. 2009) using the LD‐pruned dataset for k values 1 through 5, where k is the set number of populations. Different ADMIXTURE runs were then evaluated using the cross‐validation (CV) error rate. To ensure that read depth was not affecting population groupings, we compared read depth among clusters and found no association.

### 2.5 Genomic Differences Among Populations

To understand genomic differences between resident and anadromous salmon across the genome, we calculated the Weir and Cockerham average weighted *F*_ST_ as well as in independent 1000 bp windows, and per‐SNP weighted *F*_ST_ between each population using VCFtools (v0.1.16, Danecek et al. 2011). Miss‐assignments or individuals that likely reflect first generation migrants based on the clustering analysis (*n* = 5) were excluded from population level comparisons.

To understand how the introduced Rocky River anadromous salmon population is admixing with or resisting gene flow from the Rocky River resident population at the genomic scale, we calculated an introgression‐divergence metric by taking the difference of the windowed per‐SNP *F*_ST_ from the Rocky River resident vs. Little Salmonier River comparison and the Rocky River resident versus Rocky River anadromous comparison. This approach highlights the unique advantages of this study system in that we can use the source population for the introduction, the Little Salmonier River, as a genetic benchmark to highlight potential introgression from the Rocky River resident into the anadromous population at a fine scale across the genome. While this approach is exploratory and cannot mechanistically link any observed genetic changes to introgression specifically, it has the advantage of highlighting directional genetic change either towards or away from the source and sympatric resident populations at a fine scale across the genome. At loci where the introduced Rocky River anadromous population remains genetically identical to the Little Salmonier River population, the introgression‐divergence metric would equal zero. However, at loci where the Rocky River anadromous and resident populations have become more similar, likely as a result of gene flow, this metric will be positive. Conversely, at loci where the Rocky River anadromous population has diverged from the residents since their introduction, this metric will be negative. Windowed per‐SNP *F*_ST_ and the introgression‐divergence metric were visualized as Manhattan plots using the package ggman (https://github.com/drveera/ggman). We evaluated whether highly introgressed and divergent SNPs in the Rocky River anadromous population are associated with specific biological functions using Gene Ontology (GO) enrichment analysis. We selected the 1% most introgressed (introgression‐divergence metric > 1) and divergent (introgression‐divergence metric < 1) SNPs in R and joined them to a file containing Atlantic salmon gene names and genomic positions (extracted from the reference genome) using the intersect command in bedtools. We then copied this list into ShinyGO (Ge et al. 2020), ran the GO enrichment analysis and downloaded the top Kyoto Encyclopedia of Genes and Genomes (KEGG) pathways associated with both sets of SNPs for visualization.

### 2.6 Impact of Gene Flow on Migratory Survival

To understand the potential fitness consequences of introgression through a reduction in the marine survival of anadromous salmon in the Rocky River, we compared the resident ancestry in outgoing smolts and return migrating adults using frequency plots for the two migratory cohorts in our data (2022–2023 and 2023–2024). While Atlantic Salmon may spend either one winter or multiple winters at sea, the vast majority of the anadromous salmon in the Rocky River are 1 sea‐winter fish (Bourgeois 1998), so we are unlikely to be incurring any bias by only making comparisons across successive years. Resident ancestry was estimated using ADMIXTURE analysis by taking each individual's level of ancestry derived from the population primarily associated with residents in the *k* = 3 run. While the *k* = 3 and *k* = 4 ADMIXTURE runs were similarly well supported (Table S2), we used the resident ancestry from the *k* = 3 run because it classified the resident salmon as a single population and we were interested in the effects of introgression from the residents more broadly rather than evaluating any distinct impacts from introgression from specific subpopulations within the watershed. We combined the resident ancestry data of both migratory cohorts and statistically evaluated whether resident ancestry was higher in out migrating smolts versus returning adults using a one‐sided Wilcoxon rank‐sum test. Cohorts were pooled to increase the overall sample size (Table S1). A one‐sided test was used as we predicted that greater resident ancestry would reduce marine survival due to the contribution of maladaptive alleles suited to a resident life history. As before, we selected the top 1% and top 50 most divergent SNPs in R and joined them to a file containing Atlantic salmon gene names and genomic positions using the intersect command in bedtools. We then copied these lists into ShinyGO (Ge et al. 2020) and ran the GO enrichment analysis. The R tidyverse package (Wickham et al. 2019) was used throughout for data manipulation and visualization unless otherwise specified.

## 3 Results

### 3.1 Characterizing Genetic Variation Among Populations

**FIGURE 2:**
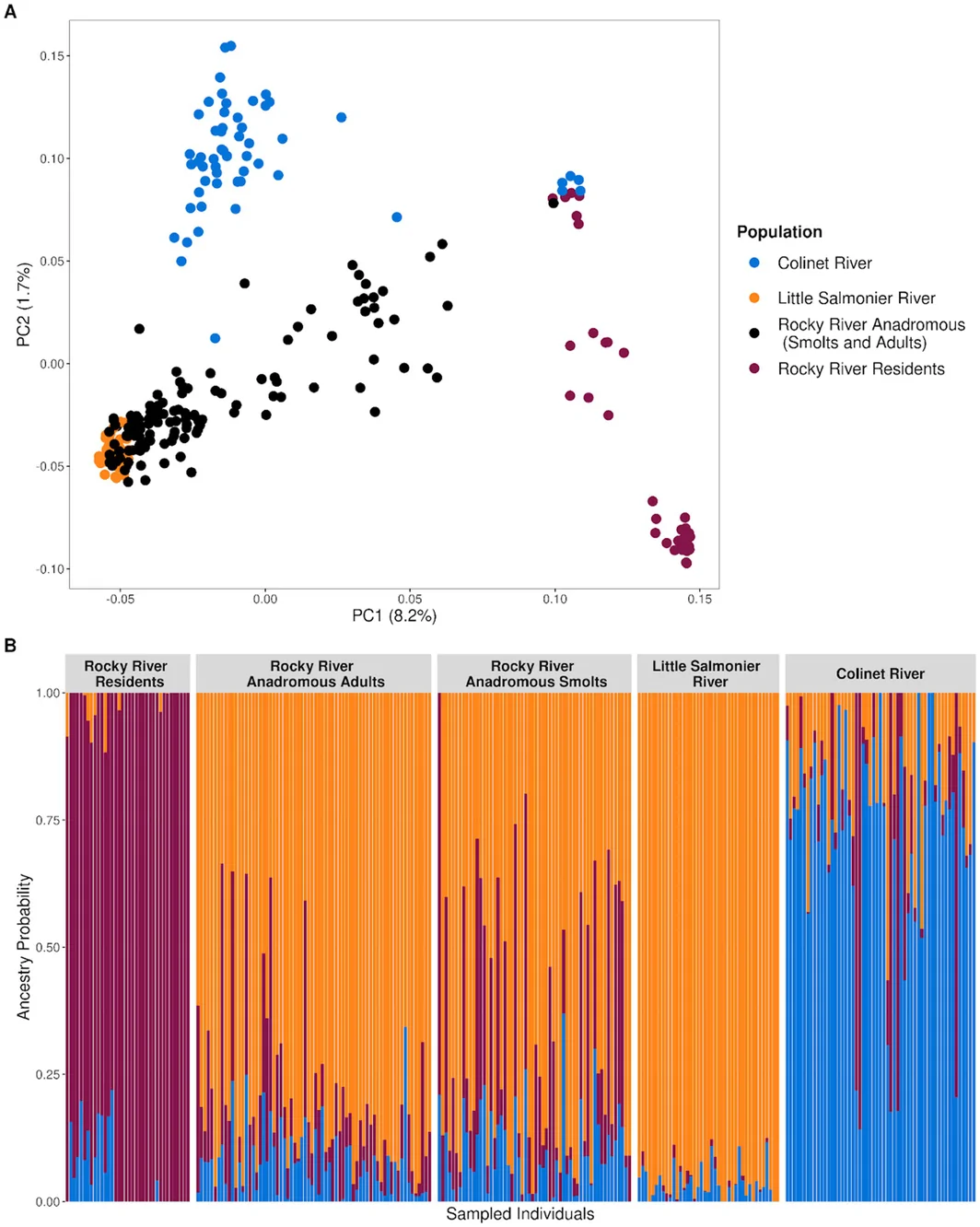
Genetic structure of anadromous and resident Atlantic salmon populations in St. Mary's Bay, Newfoundland, Canada, inferred using Principal Components Analysis and ADMIXTURE including 256 individuals and 5,652,164 single‐nucleotide polymorphisms. (A) Scatterplot of PCA results; (B) individual ADMIXTURE estimates (k = 3).

The final whole‐genome dataset contained 256 individuals genotyped at ~4× coverage across 5,652,164 SNPs with minor allele frequency > 0.01. A PCA of the whole‐genome sequencing data from all the samples included in the study revealed distinct population groups, with samples from Little Salmonier River, Colinet River, Rocky River anadromous and Rocky River residents each broadly clustering in separate groups (Figure 2). PC1, explaining 8.2% of the variation in the genomic data, corresponded to the anadromous‐resident life history axis, with all resident individuals being grouped on one end of PC1 and the anadromous individuals grouping towards the opposite end. The introduced Rocky River anadromous population show evidence of admixture with the Rocky River resident population, occupying a distinct space on the PC1 axis between their source population, Little Salmonier River, and the Rocky River residents. ADMIXTURE analysis largely concurred with the PCA, grouping the Rocky River residents, Little Salmonier River and Colinet River as distinct populations (Figure 2). The Rocky River anadromous group was not assigned its own population and was instead represented as an admixed group of the other populations, showing the highest levels of ancestry from the Little Salmonier population but also substantial introgression from the Rocky River resident population. The best supported k‐values were *k* = 3 and *k* = 4, with the only difference being that ADMIXTURE split the Rocky River residents into two distinct populations in the *k* = 4 run (Figure S1). In the Rocky River, migratory individuals (i.e., smolts and returning adults) appeared to almost exclusively come from the admixed Rocky River anadromous population, except for 1 smolt that came from the resident population. We also found limited evidence of straying between the Rocky and Colinet Rivers, with small numbers of individuals from the Colinet River group clustering closely with the Rocky River anadromous (*n* = 2) and Rocky River resident (*n* = 5) populations rather than with the main Colinet River group.

### 3.2 Genomic Differences Among Populations

**FIGURE 3:**
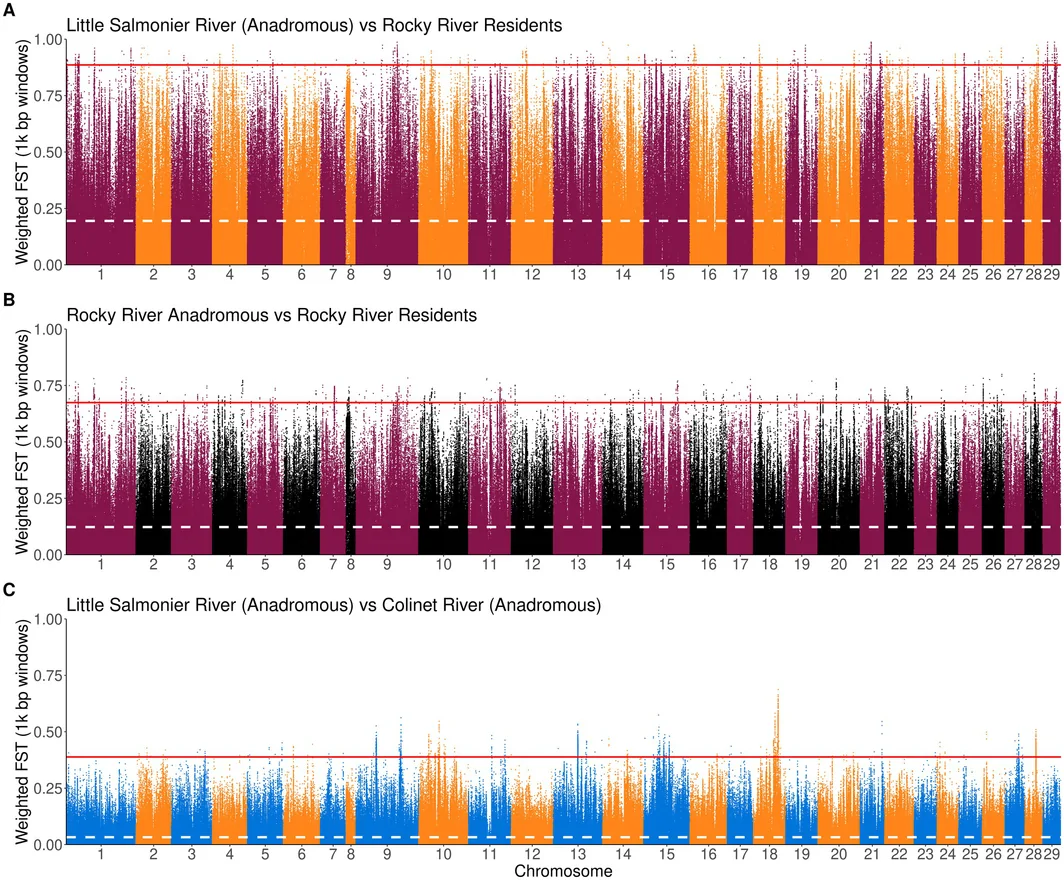
Genomic differentiation between pairs of anadromous and resident Atlantic salmon populations in St. Mary's Bay, Newfoundland, Canada. Manhattan plots show F_ST_ for 1000 base pair windows across the genomes for pairwise comparisons of several focal populations. (A) Comparison of Little Salmonier River population and Rocky River resident population; (B) comparison of Rocky River anadromous population and Rocky River resident population; (C) comparison of Little Salmonier River population and Colinet River population. The top two plots (A, B) compare two distinct anadromous populations to a resident population, while the bottom plot (C) shows a comparison of two anadromous populations (Little Salmonier and Colinet Rivers). Dotted white lines show population‐level weighted *F*_ST_ and red lines show the top 0.1% *F*_ST_ value for that comparison.

Pairwise *F*_ST_ comparisons revealed that anadromous and resident populations in our study were substantially more genetically different than anadromous populations were from each other (Figure 3; Figure S2). When compared with the Rocky River residents, the Little Salmonier River, Colinet River, and Rocky River anadromous populations showed a weighted *F*_ST_ of 0.2, 0.14, and 0.12 respectively. In contrast, the Little Salmonier and Colinet River populations (both anadromous) had a weighted *F*_ST_ of 0.033 when compared with each other. The Little Salmonier and introduced Rocky River anadromous population showed a weighted *F*_ST_ of 0.013. Per‐locus windowed *F*_ST_ comparisons reveal larger peaks and overall higher *F*_ST_ across the genome in comparisons between resident and anadromous populations, suggesting a role for both adaptation and genetic drift in driving the high levels of genetic differentiation.

### 3.3 Introgression and Divergence Across Rocky River Anadromous Genome

**FIGURE 4:**
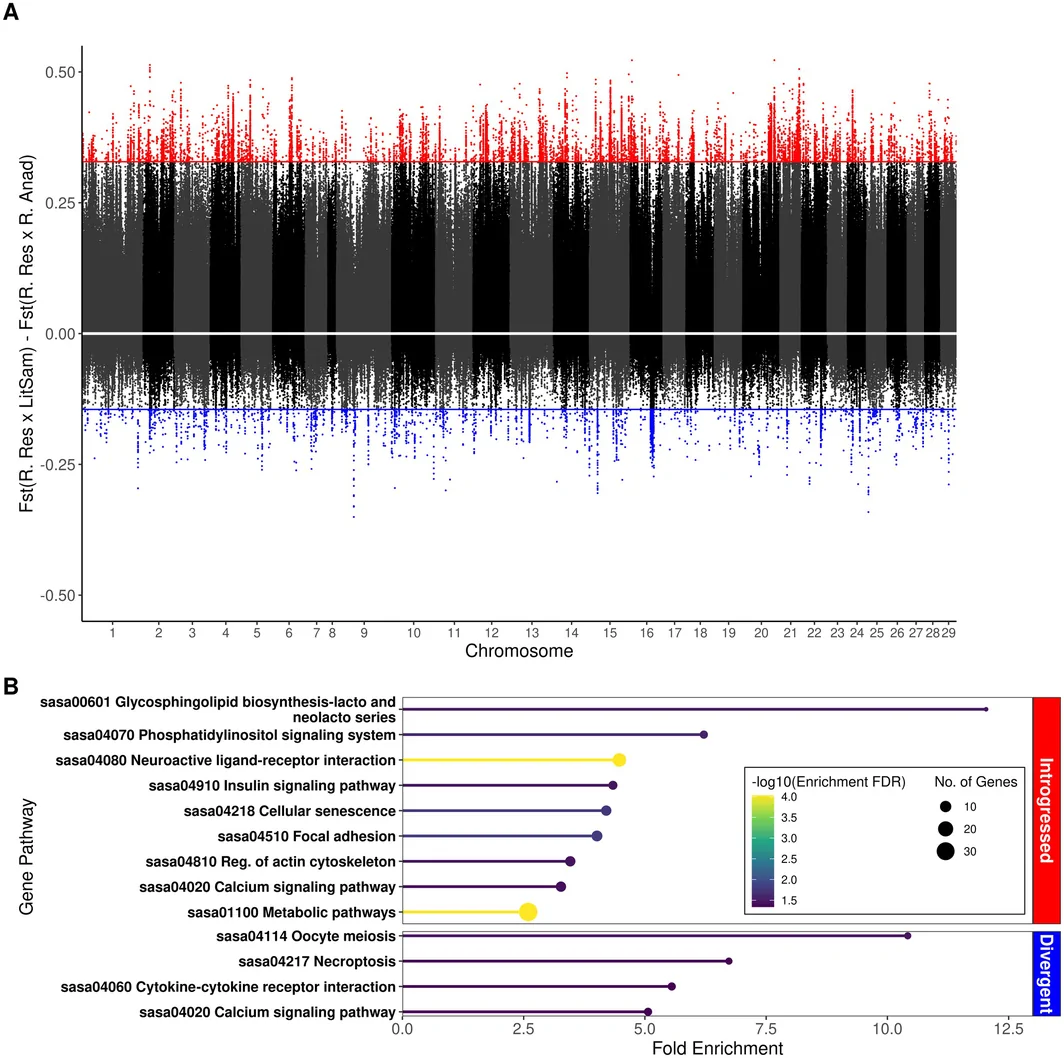
Genomic introgression and divergence across the genome for the introduced Rocky River anadromous population. (A) Manhattan plot showing an introgression‐divergence metric for 1000 base‐pair windows across the Rocky River anadromous population genome. The introgression‐divergence metric is calculated by taking the difference in F_ST_ from the Little Salmonier (source population) (LitSam) **×** Rocky River resident population (R. Res) comparison and the Rocky River anadromous (introduced population) (R. Anad) **×** Rocky River resident (R. Res) comparison. A value of zero for this metric indicates that the Rocky River anadromous population is unchanged genetically at that locus since their introduction, while a positive value indicates introgression from the resident population and a negative value indicates divergence from the resident population. Red and blue dots show the top introgressed and divergent loci, which were used for gene ontology enrichment analysis. Thresholds were set at the highest and lowest 1% of the introgressed (values > 1) and divergent (values < 1) loci. (B) Results from gene ontology (GO) enrichment analysis (Ge et al. 2020) showing Kyoto Encyclopedia of Gene and Genomes (KEGG) pathways significantly associated with introgressed and divergent loci outliers.

PCA and ADMIXTURE results revealed evidence of substantial introgression from the resident population into the anadromous population in the Rocky River despite distinct clustering of anadromous and resident populations. We used a novel comparison of per‐locus *F*_ST_ values to evaluate which regions in the anadromous genome were introgressing freely and which were resisting introgression. This introgression‐divergence metric varied substantially across the Rocky River anadromous salmon genome (Figure 4). Most loci showed positive values for this metric (68% of loci were greater than 0.01), suggesting that most regions of the genome show introgression from the resident population. However, many regions show values near zero (19% of loci were between 0.01 and −0.01), suggesting these regions are resisting gene flow, and some regions even show negative values (13% of loci were less than −0.01), suggesting that these regions are diverging away from the resident genome despite gene flow. Highly introgressed and divergent loci were enriched for 9 and 4 KEGG pathways respectively (Figure 4). Pathways associated with the highly divergent loci, which may represent traits important to the anadromous life history, included the calcium‐signalling pathway, cytokine‐cytokine receptor interaction, necroptosis and oocyte meiosis.

### 3.4 Selection Against Resident Ancestry in Migrating Salmon

**FIGURE 5:**
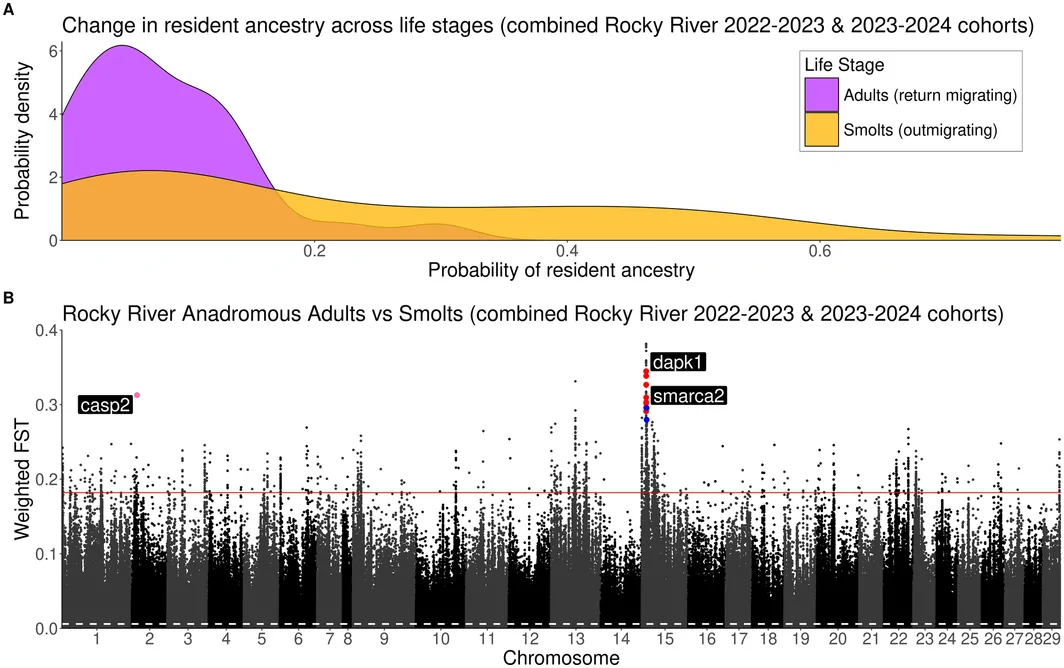
Evidence of selection against resident ancestry during marine migration for Rocky River anadromous salmon using data from both migratory cohorts from the sampling period (2022 smolts → 2023 adults; 2023 smolts → 2024 adults). (A) Frequency plot showing the probability of resident ancestry, extracted from ADMIXTURE analysis, in out migrating smolts and returning adult anadromous salmon. Resident ancestry differed significantly between smolts and adults (One‐sided Wilcoxon rank sum test, *w* = 654.5, *p* = 0.006**). (B) Manhattan plots showing *F*_ST_ for 1000 base pair windows across the genomes for comparison between out migrating smolts and returning adults. The red line shows the top 0.1% of *F*_ST_ and the dashed white line shows genome‐wide weighted *F*_ST_.

To explore the implications of introgression and resident ancestry in individuals displaying a migratory phenotype, we compared out migrating smolts to returning adults across two cohorts. We observed significant differences in resident ancestry between out migrating smolts (mean = 0.23, SD = 0.21, *n* = 34) and returning adult anadromous salmon (mean = 0.081, SD = 0.069, *n* = 28) from the Rocky River anadromous population across the combined migratory cohorts (one‐sided Wilcoxon rank‐sum test, *w* = 654.5, *p* = 0.006), suggesting that higher resident ancestry reduces marine survival in this population (Figure 5). A *F*_ST_ comparison between smolts and adults (weighted *F*_ST_ = 0.0023) revealed several distinct *F*_ST_ peaks suggesting regions under selection. ShinyGO analysis found significant enrichment for the top 50 most divergent loci. Only 3 genes of known function (*dapk1, smarca2*, and *casp1*) could be associated with the top 50 most divergent loci; therefore, we present and discuss these genes directly rather than their KEGG pathways obtained from ShinyGO (Figure 5). *dapk1* and *smarca2* are located on the same divergent region of chromosome 15, while *casp1* is located on chromosome 2.

## 4 Discussion

Intraspecific diversity can have important influences on how species adapt to changing environments (Des Roches et al. 2018, 2021). Alternative migratory strategies are a clear example of intraspecific life history variation that can be associated with substantial genetic differences, yet the forces that maintain these differences are often unclear. In this study, we assessed how genetic differences associated with migratory strategy may affect gene flow in Atlantic Salmon ( *Salmo salar* ) in a natural experiment where a migratory barrier was removed and migratory and non‐migratory salmon have been brought into contact. Our results demonstrate that migratory and resident non‐migratory populations can display significant genome‐wide genetic divergence following colonization after the last ice age, likely as a result of both genetic drift and adaptation. However, despite the substantial genetic divergence, we find evidence of unidirectional introgression between migratory morphs following secondary contact. Our genome‐wide exploration of introgression suggests that some regions of the genome resist introgression, and our comparison of ancestry across smolts and adults revealed evidence of a fitness cost caused by outbreeding depression associated with the marine migratory stage. These findings build on a growing body of studies exploring the genomic basis of migratory phenotype in salmonids (Perrier et al. 2013; Zueva et al. 2014; Kjærner‐Semb et al. 2021; Tigano and Russello 2022; Kess et al. 2024; Beck et al. 2025; Lehnert et al. 2025) and provide a perhaps novel examination of the potential cost of introgression among resident and anadromous morphs. These results highlight potential implications from salmonid enhancement activities, including significant impacts to resident populations from stocking with anadromous fish and the potential for introgression from resident populations to reduce the productivity of anadromous populations through outbreeding depression.

The comparison of the resident and migratory forms revealed remarkably high genome‐wide genetic divergence between the residents and all anadromous populations in our study despite close geographic proximity, likelihood of common ancestry following the last glacial period and the potential for unidirectional geneflow even prior to the removal of the migratory barrier. This divergence was evident at most loci but intensified into peaks at many loci distributed across the genome. In contrast, the two anadromous populations showed a much lower degree of genome‐wide genetic divergence and fewer and less intense divergent peaks. Taken together, the results are consistent with a hypothesis of both widespread genetic drift and selection for migratory phenotype acting on a large number of loci across the genome in this system. Previous genetic studies on proximate resident and anadromous Atlantic salmon populations report comparable, or greater, genetic divergence associated with migratory strategy (Perrier et al. 2013; Zueva et al. 2014; Kjærner‐Semb et al. 2021; Harder and Christie 2022; Hindar et al. 2025). Many of these studies highlight the increased intensity of genetic drift in resident populations, driven by the tendency of these populations to be smaller and more isolated from gene flow (Tonteri et al. 2007; Perrier et al. 2013; Adams et al. 2016; Subramanian and Kumar 2023). Additionally, many resident salmon populations show evidence of parallel evolution, consistent with the hypothesis that the resident migratory strategy itself can result in altered selection on certain traits (Perrier et al. 2013; Zueva et al. 2014; Kjærner‐Semb et al. 2021; Harder and Christie 2022). Therefore, while our results largely support the established notion that strong genetic divergence between anadromous and resident Atlantic salmon populations is driven by a combination of genetic drift and selection, we highlight the importance that selection plays in this process by acting strongly on a large number of loci.

Our results also indicate that substantial introgression from the native resident into the introduced anadromous morph has occurred in the Rocky River over the decades since the introduction and an absence of strong reproductive barriers has allowed this gene flow between the morphs. While Rocky River anadromous individuals varied in their level of resident introgression, two‐thirds show at least some level of resident ancestry. Elsewhere, studies have explored the potential for gene flow among sympatric resident and migratory morphs of Atlantic salmon: some morphs appear to be genetically distinct and reproductively isolated (Verspoor and Cole 1989; Birt et al. 1991a), while in other populations resident and anadromous individuals appear to be part of the same gene pool (Verspoor and Cole 1989; Adams et al. 2016). Contrasting with these previous studies, the Rocky River populations appear to show both strong genetic divergence and ongoing gene flow. To our knowledge, such a situation has only been reported in three locations: the Rocky River and the Northwest River (Adams et al. 2016) in Newfoundland, Canada and the River Namsen, Norway (Hindar et al. 2025). In all cases, the now‐sympatric resident and anadromous salmon populations were historically separated by migratory barriers that were removed as part of salmon enhancement efforts. Because these populations have originally evolved allopatrically, there would have been no selective pressure to develop reproductive barriers that would maintain adaptive differences (Nosil et al. 2003). We suggest that the lack of reproductive barriers between these populations with substantial genetic and life history differences is a function of the artificial nature of and short time since secondary contact. Over time and depending on the strength of divergent selection, these populations may either develop mechanisms to prevent gene flow or be subsumed into a single, genetically admixed population (Hendry 2009).

This introduction and the subsequent observed introgression among migratory forms provide a unique opportunity to explore the potential for outbreeding depression of admixed individuals. Here, multiple lines of evidence suggest that gene flow from the Rocky River residents into the anadromous population is likely having a fitness cost caused by outbreeding depression. First, migrating individuals with higher resident ancestry seem to have lower marine survival and/or higher straying rates. Second, out migrating smolts show substantial genetic differences from returning adults, with several loci putatively under strong selection. Third, one‐third of loci in the Rocky River anadromous genome show resistance to introgression from resident genetics despite obvious gene flow, suggesting that there is strong selection to maintain these loci in their ancestral state. Outbreeding depression occurs when individuals from separate populations breed and their offspring have reduced fitness due to the dilution of local adaptation or the breakup of coadapted genes (Edmands and Timmerman 2003; Frankham et al. 2011). This is likely when populations inhabit different environments (leading to adaptive differentiation) and have been isolated for longer than 500 years. Notably, genetic drift alone is not expected to be a strong driver of outbreeding depression (Frankham et al. 2011). The Rocky River anadromous and resident populations inhabit distinct environments for much of their life cycles and have likely been isolated for several thousand years prior to secondary contact. Therefore, hybrids may possess alleles that leave them poorly suited for ocean migration, explaining their reduced migratory survival. Outbreeding depression has been previously demonstrated to result from breeding between distinct ecotypes in other species. For example, hybrid offspring from odd‐ and even‐year migratory cycles of Pink Salmon ( *Oncorhynchus gorbuscha* ) (Walbaum, 1792) also show reduced marine survival (Gharrett et al. 1999; Gilk et al. 2004). Similarly, the establishment of a new migratory range in Eurasian Blackcaps ( *Sylvia atricapilla* ) (Linnaeus, 1758) has led to strong genetic divergence, apparent reproductive isolation and adaptive differentiation putatively maintained by outbreeding depression in less than 30 years (Rolshausen et al. 2009). In Three‐Spined Stickleback ( *Gasterosteus aculeatus* ) (Linnaeus, 1758), hybrids between benthic and limnetic morphs are less well adapted to their respective environments (Rundle 2002). However, the apparent outbreeding depression we report here is, to our knowledge, a unique demonstration of this effect between resident and migratory morphs.

While our results are consistent with reduced marine survival of admixed individuals caused by outbreeding depression, other mechanisms could have contributed to the same patterns. For instance, the reduction in resident ancestry between smolts and returning adults might not arise from mortality at sea, but instead from an increase in straying among rivers brought about by less precise natal homing. However, this in itself could be considered outbreeding depression as rates of straying are typically low in Atlantic Salmon (Jonsson et al. 2003; Keefer and Caudill 2014) and salmon populations from different rivers tend to be genetically distinct and locally adapted (Dionne et al. 2008; Zueva et al. 2021; Lecomte et al. 2024). Thus, strays tend to have notably reduced reproductive success relative to native individuals (Mobley et al. 2019), suggesting that increases in straying rates would generally be maladaptive. Strong genetic drift caused by a founder effect could also contribute to divergence between an introduced population and its source population, potentially confounding evidence for introgression. However, the founding gene pool for the anadromous Rocky River population was large, involving 1040 adult salmon over 4 years resulting in a release of an average of 333,980 fry per year. Moreover, genetic drift is unlikely to have caused patterns resembling significant, genome‐wide introgression from the sympatric resident population as it is non‐directional and anadromous and resident Atlantic Salmon are known to interbreed when they come into contact (Adams et al. 2016; Hutchings et al. 2019; Hindar et al. 2025). Given the strong likelihood of continued introgression over several generations, genetic drift alone is unlikely to account for those regions of the genome that remain completely unintrogressed. Stochastic events in the marine environment could also mimic selection but would likely result in large variations in resident ancestry across migratory cohorts, which does not occur in our dataset (Table S1, Figure S3). Viability selection could partially account for the signatures of putative selection observed in the genome‐wide *F*_st_ analysis comparing smolts with returning anadromous adults, although the concurrent decline in resident ancestry over the same period suggests that some portion of the observed genetic differences are due to selection acting against resident introgression. While our analyses are limited in their ability to precisely apportion the relative effects of outbreeding depression, genetic drift, or other forms of selection in causing the observed declines in resident ancestry during the marine migration and the associated genomic patterns, our results suggest that outbreeding depression, in the form of increased at‐sea mortality or higher straying rates, is the likely primary driver.

The outbreeding depression we likely observed between the resident and anadromous Rocky River salmon may be a result of key adaptive differences stemming from their distinct migratory phenotypes. For example, we observed that the Rocky River anadromous genes that are resisting resident introgression were enriched for the calcium signalling pathway, suggesting that this pathway is under selection to be maintained in its ancestral, anadromous state. Calcium signalling is critical to triggering the physiological response to osmotic stress in salmonids and other fish (Nearing et al. 2002; Fiol and Kültz 2007; Papakostas et al. 2012) and reduced saltwater tolerance has been repeatedly demonstrated in several resident Atlantic Salmon populations (Birt et al. 1991a; Nilsen et al. 2003, 2007; Piironen et al. 2013). Indeed, Zueva et al. (2014) also linked calcium signalling to salinity adaptation in a range of saltwater‐, brackish‐, and freshwater‐inhabiting Atlantic Salmon populations. If Rocky River resident individuals have a reduced ability to detect and respond to osmotic stress, then the observed introgression into anadromous individuals could reduce the ability of hybrids to make appropriate physiological responses to saltwater.

We also observed that the necroptosis pathway was enriched in those same introgression‐resistant genes, and that two of the loci under the strongest putative selection (*dapk1* and *casp2*) during marine migration in the anadromous population are associated with apoptosis (Bouchier‐Hayes and Green 2012; Zhang et al. 2025). Both the necroptosis and apoptosis pathways serve important roles in the immune system, the former related to pathogen defence and the latter to tumour suppression. Immune function may represent a critical adaptive difference between resident and anadromous salmon because they inhabit distinct environments that have different suites of parasites (Bakke and Harris 1998). Indeed, resident and anadromous salmon populations can vary dramatically in their tolerance to certain parasites (Zueva et al. 2014). They can also differ in their immune response during smoltification (Ingerslev et al. 2006; Lemmetyinen et al. 2013), which is the intense physiological process that prepares a juvenile salmon to migrate to the ocean and involves substantial changes to immune function (Johansson et al. 2016; Krasnov et al. 2024). During this period, they are particularly vulnerable to disease (Johansson et al. 2016). We recognize that caution must be used in creating narratives around the interpretation of the functional traits associated with outlier loci due to the complex networks of interaction among genes (Pavlidis et al. 2012). However, it is notable that both saltwater tolerance and immune function have been previously demonstrated as divergent traits between anadromous and resident populations. Therefore, we add to the body of evidence suggesting that these may be consistent divergent adaptations that evolve in resident Atlantic Salmon populations and suggest that they may play a role in driving the outbreeding depression we observed.

Surprisingly, all admixed individuals we sampled in the Rocky River expressed a migratory life history and we found little evidence for introgression from the anadromous into the resident population. This asymmetry in the distribution of ancestry values suggests that migratory strategy has a strong genetic basis, with anadromy being dominant (e.g., Hindar et al. 2025), and that some mechanism is preventing bidirectional gene flow. We propose two possible mechanisms: (1) hybrids adopt the anadromous migratory strategy and may be more likely to breed with other anadromous individuals, or (2) severe outbreeding depression prevents hybrid residents from reaching adulthood. Additional juvenile sampling will be required to explore these mechanisms by evaluating spatial differences in spawning and the ancestry values from a larger sample of resident individuals, as it is possible that some hybrids follow the resident migratory strategy but that we failed to capture them. Nonetheless, our data do allow us to make robust conclusions about the degree of genetic differentiation and gene flow among populations and the impact of potential outbreeding depression on the marine migratory stage.

Range‐wide declines in Atlantic salmon populations (Lehnert et al. 2019) have increasingly prompted interest in using stocking as a conservation tool (Lennox et al. 2021). This study highlights the potential pitfalls of stocking and salmon enhancement activities when they do not properly consider the intraspecific variation associated with alternative migratory strategies. Based on available habitat, the Rocky River was expected to support a run of 2000–4000 anadromous salmon but has never come close to meeting this target (Bourgeois 1998), with numbers of returning adults dropping into the low hundreds in recent years (DFO 2025). Our study design does not allow us to quantitatively assess the demographic impact of the outbreeding depression we observed, but it is not unreasonable to suggest that interbreeding with the abundant resident salmon in this watershed could have played a role in the low productivity and eventual decline of the Rocky River anadromous population. This would not be without precedent; a similar enhancement project involving the removal of a migratory barrier and subsequent secondary contact of resident and anadromous Atlantic salmon in the River Namsen, Norway has resulted in the near genetic extinction of the resident population following extensive introgression (Hindar et al. 2025). Notably, in both Hindar et al. (2025) and the present study, it appears that hybrids exhibit an anadromous life history. Thus, interbreeding may hinder the success of any enhancement projects aimed at boosting anadromous populations as outbreeding depression may reduce the survival of hybrids and also threaten the persistence of genetically unique resident populations through extinction‐by‐hybridization, as hybrids tend to become anadromous. While it is clear that genetically distinct resident and anadromous salmon populations can coexist in nature where they have evolved in sympatry (Verspoor and Cole 1989; Birt et al. 1991a, 1991b), these migratory morphs may constitute genetic threats to each other when they have no history of coevolution and human activities bring them into contact.

Many migratory salmonid species that hold substantial ecological, cultural, and economic importance face serious threats (Lehnert et al. 2019; Wilson et al. 2022; Dadswell et al. 2022; Kaeriyama 2023). These species have unique conservation challenges because they often form genetically distinct populations associated with different migratory modes (Dodson et al. 2013; Willis et al. 2021). Resolving the intraspecific variation associated with these populations is critical for defining conservation units and understanding the impacts of human activities that can bring populations into contact. We investigated this intraspecific variation in resident and anadromous Atlantic salmon populations in Newfoundland, Canada, and show that, under certain conditions, migratory strategy can drive substantial genetic divergence, including key adaptive differences. These adaptive differences appear to result in a fitness cost through outbreeding depression when previously allopatric migratory morphs are brought into contact by human activities. Our results clarify the role that migratory strategy plays in driving adaptive divergence and show that stocking and enhancement activities that ignore this variation may be hamstrung by maladaptive gene flow.

## Funding

This work was supported by Foundation for Conservation of Atlantic Salmon in partnership with SAEN (grant number NL‐2024‐07), as well as NSERC Discovery (grant number RGPIN‐2023‐05864), Memorial University of Newfoundland, and Fisheries and Oceans Canada.

## Ethics Statement

Animal handling procedures were approved by the Memorial University of Newfoundland Institutional Animal Care Committee (IACC) following Canadian Council of Animal Care (CCAC) guidelines, under protocol 24‐01‐IF.

## Conflicts of Interest

The authors declare no conflicts of interest.

## Supporting information


**Figure S1:** Individual ADMIXTURE estimates (k = 4) of anadromous and resident Atlantic salmon populations in St. Mary's Bay, Newfoundland, Canada using an LD‐pruned version of a dataset containing 256 individuals and 5,652,164 single‐nucleotide polymorphisms.
**Figure S2:** Genomic differentiation between pairs of anadromous and resident Atlantic salmon populations in St. Mary's Bay, Newfoundland, Canada. Manhattan plots show F_ST_ for 1000 base pair windows across the genomes for pairwise comparisons of populations not included in Figure 3. (A) Comparison of Little Salmonier River population and Rocky River anadromous population (average weighted *F*_ST_ = 0.013); (B) comparison of Rocky River anadromous population and Colinet River population (average weighted *F*_ST_ = 0.019); (C) comparison of Rocky River resident population and Colinet River population (average weighted *F*_ST_ = 0.14). Dotted white lines show population‐level weighted F_ST_ and red lines show the top 0.1% *F*_ST_ value for that comparison.
**Figure S3:** Frequency plots showing evidence of selection against resident ancestry during marine migration for Rocky River anadromous salmon using data from both migratory cohorts from the sampling period (2022 smolts → 2023 adults; 2023 smolts → 2024 adults). Probability of resident ancestry extracted from ADMIXTURE analysis.
**Figure S4:** Estimated allele frequencies, PC1, and PC2 scores using imputed and maximum likelihood (PCANGSD) genotyping approaches for chromosome 28 of the genomic samples in our dataset. All three metrics were > 99% correlated (*p* = −2.2e16).
**Table S1:** Resident ancestry values and sample size for migratory cohorts used to evaluate the impact of resident ancestry on marine survival in Rocky River anadromous salmon.
**Table S2:** Cross‐validation (CV) error rates for ADMIXTURE runs *k* = 1 through *k* = 5.

## Data Availability

Raw whole‐genome sequencing reads will be made available at the European Nucleotide Archive, which issues its own DOIs and Project Numbers. Metadata will be made available at the Federated Research Data Archive, which issues its own DOIs. Data has not been uploaded yet but will be made available should the manuscript be accepted.
