# Supplementary material for: Introgression and Potential Outbreeding Depression Following Anthropogenically‐Induced Secondary Contact Between Anadromous and Resident Atlantic Salmon ( Salmo salar )
Source: Evol Appl. 2026 Sep 25;19(9):e70318. doi: 10.1111/eva.70318 (PMC13615306; doi:10.1111/eva.70318)
Supplement: Supplementary file 1 [file EVA-19-e70318-s001.docx]

**Supporting Information**

**Figure S1.** Individual ADMIXTURE estimates (k=4) of anadromous and resident Atlantic salmon populations in St. Mary’s Bay, Newfoundland, Canada using an LD-pruned version of a dataset containing 256 individuals and 5,652,164 single-nucleotide polymorphisms.

**Figure S2.** Genomic differentiation between pairs of anadromous and resident Atlantic salmon populations in St. Mary’s Bay, Newfoundland, Canada. Manhattan plots show F_ST_ for 1000 base pair windows across the genomes for pairwise comparisons of populations not included in Fig. 3. A) Comparison of Little Salmonier River population and Rocky River anadromous population (average weighted F_ST_ = 0.013); B) comparison of Rocky River anadromous population and Colinet River population (average weighted F_ST_ = 0.019); C) comparison of Rocky River resident population and Colinet River population (average weighted F_ST_ = 0.14). Dotted white lines show population-level weighted F_ST_ and red lines show the top 0.1% F_ST_ value for that comparison.

**Figure S3.** Frequency plots showing evidence of selection against resident ancestry during marine migration for Rocky River anadromous salmon using data from both migratory cohorts from the sampling period (2022 smolts → 2023 adults; 2023 smolts → 2024 adults). Probability of resident ancestry extracted from ADMIXTURE analysis.

**Figure S4.** Estimated allele frequencies, PC1, and PC2 scores using imputed and maximum likelihood (PCANGSD) genotyping approaches for chromosome 28 of the genomic samples in our dataset. All three metrics were >99% correlated (p = -2.2e16).

**Table S1.** Resident ancestry values and sample size for migratory cohorts used to evaluate the impact of resident ancestry on marine survival in Rocky River anadromous salmon.

| **Migratory Cohort** | **Smolt Resident Ancestry (mean (SD))** | **Adult Resident Ancestry (mean (SD))** | **# Smolts** | **# Adults** |
| --- | --- | --- | --- | --- |
| 2022 → 2023 | 0.26 (0.22) | 0.07 (0.07) | 18 | 20 |
| 2023 → 2024 | 0.19 (0.19) | 0.10 (0.06) | 16 | 8 |

**Table S2.** Cross-validation (CV) error rates for ADMIXTURE runs k = 1 through k = 5.

| **K** | **CV Error Rate** |
| --- | --- |
| 1 | 0.52998 |
| 2 | 0.4897 |
| 3 | 0.48663 |
| 4 | 0.48639 |
| 5 | 0.48788 |
